# The Hospital World

**Published:** 1911-04-01

**Authors:** 


### The Hospital World.

### Personalia.

The Queen has become patron of St. Mary's Hospital, Paddington.

The King and Queen have become patrons of the City of London Hospitals for the diseases of the chest.

The late Mr. Bonnett, whose bequest of £200 to Addenbrooke's Hospital is recorded in another column, was for many years Secretary to this Hospital, and from January 1905 until the time of his death was legal adviser to the institution. He was succeeded by Mr. Richard J. Coles, the present superintendent.

A very distinguished hospital supporter has died at Madras in the person of Rajah Sir Savalay Ramaswamy Mudaliyar, C.I.E. Sir Ramaswamy, the head of a large business firm, built, and partly endowed, the Maternity Hospital in Madras, which is named after him, and he also provided a ward at the Conjeeveram Hospital and a dispensary at Cuddalore. He was created a C.I.E. in 1885, and was knighted at the first Jubilee of Queen Victoria. The title of Rajah was bestowed upon him as a personal distinction. He was one of the first merchant princes in India to take a prominent part in the development of hospitals, and his example has been a valuable stimulant to others.

The following tribute to Mr. H. O. Williams, Hon. Secretary of the Luton Bute Hospital, was paid at the recent annual meeting by the Chairman, Mr. W. R. Phillips, who said that for many years it had always been a great pleasure to be associated with Mr. Williams, who was always most thorough in all his work. Before any important step was taken, Mr. Williams always investigated every point, and brought the result to the committee. Few of them would credit the exceedingly careful and neat way in which he kept the books; he always was up to date and up to time. Mr. W. Austin said the committee appreciated very highly all that Mr. Williams did. His accuracy and punctuality was marvellous. The hospital was immensely indebted to him for the time, care, and ability he bestowed upon it. Dr. Doubble and Mr. Warren also warmly congratulated Mr. Williams on his work.

The following interesting record is taken from the annual report of the Bristol General Hospital, which was issued last week: "During the year the hospital has lost several highly-honoured and much-valued friends. In February Sir Edward Payson Wills, Bart., passed away at a ripe age. He had been a member of the committee since 1897. In August, at the advanced age of eighty-nine, occurred the death of Mr. Coe, who in former years served the surgical staff of the hospital with great distinction. In November Dr. Charles Wooliston Belfield died, who at one time was a student at the hospital. His deep interest in its work is evidenced by the munificent legacy of £5000 bequeathed by him to the institution. In December the sad news was received of the death of Mrs. Proctor Baker. Her interest in the work of the General Hospital had never ceased since her association with it, through her husband, the late Alderman W. Proctor Baker. In his capacity as president he was the wise and sympathetic guide and counsellor of the hospital, and it was during his presidency that it made such marked progress in the medical world. It was only a few months before her death that Mrs. Proctor Baker gave £10,000 to the hospital in memory of her husband. Both Mr. and Mrs. Proctor Baker had taken a great interest in the work of the Maternity Department, and this generous gift has made it possible, as well as incumbent upon the committee, to embark upon the scheme for making this much-needed addition to the present equipment of the hospital."

(It is hoped to include a maternity ward in the new wing which will be built as a memorial to King Edward.)

### Elections and Appointments.

The Duchess of Portland has been elected President of the Nottingham Hospital for Women.

Mr. F. Impey has been elected hon. lay secretary, and Mr. Howard Lloyd treasurer of the Birmingham Hospital Sunday Fund.

Captain the Hon. H. T. Allsopp has been appointed chairman of the House Committee of Coton Hill Hospital for the Insane, Stafford.

Mr. W. D. Forsyth, Mr. J. Jackson, Mr. J. F. Addison, Mr. B. E. Crump, Mr. A. Jagger, Mr. A. Law, Mr. E. J. Shaw, Mr. H. H. Tucker, Mr. E. F. Shedden, Mr. C. Nicklin, Mr. E. Evans, and Mr. D. Watt have been elected on the General Committee of Walsall and District Hospital.

At the annual meeting of the Hospital Saturday Fund the report for 1910 showed that the income was £34,783, or £4,121 more than in the previous year, and the total grants were £32,439. The management expenses were 7.4 per cent. of the gross receipts.

The retiring members, the Rev. Brook Deedes, Mr. Clutton Brock, Mrs. Carrington, Mr. S. Baer, and Dr. Weaver have been re-elected to the committee of the Mount Vernon Hospital for Diseases of the Chest, and the election of Mr. Percival Etheridge as a member of committee has been confirmed. Sir Herbert Cohen and Mr. H. M. Knowles have been elected life governors of the hospital.

